# Pericardiectomy for Pleuropericardial Effusion Complicating Bacterial Pneumonia

**DOI:** 10.1155/2010/715953

**Published:** 2010-05-30

**Authors:** Andrea Quarti, Fernando Maria de Benedictis, Elli Soura, Marco Pozzi

**Affiliations:** ^1^Department of Congenital and Paediatric Cardiac Surgery and Cardiology, Ospedali Riuniti, 60128 Ancona, Italy; ^2^Department of Pediatrics, Ospedali Riuniti, 60128 Ancona, Italy

## Abstract

Severe pericardial effusion is a rare complication of bacterial pneumonia and it usually disappears under medical treatment. Herein we report a case of a girl with a congenital immunodeficient syndrome and bacterial pneumonia, who developed recurrent and life-threatening pericardial effusion refractory to medical treatment. She was finally treated with pericardiectomy.

## 1. Case Report

In November 2008, a 10-year-old girl, 42 Kg, was admitted to our Institution with pneumonia. The patient suffered from congenital immunodeficient syndrome associated to neutropenia and was under treatment with monthly subcutaneous injection of immunoglobuline, discontinued in November 2007. 

Chest X-ray showed a bilateral pleural effusion and lung consolidation on the right para cardiac border. Laboratory data revealed leucocitosis (34,3 × 10^3^/mmc) with neutrophilia (75%) and increased values of CRP (24,4 mg/dL), procalcitonine (1,7 ng/mL), and NT-proBNP (3.331 pg/mL). Antibiotic therapy was started with Ceftazidim, Claritromicin, and Teicoplanin.

**Figure 1:**
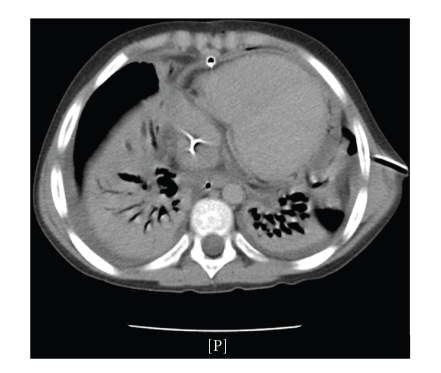
CT scan after subxifoid pericardial drainage.

A chest drain was inserted on the left side and 200 mL of yellow dense fluid was drained. An echocardiographic examination revealed a large pericardial effusion with a maximum extent of 22 mm and signs of cardiac tamponade. A 22 ch. pericardial drain was inserted through a subxifoid approach and 450 mL of limpid fluid was drained. Neither the pleural nor the pericardial fluid demonstrated bacterial growth but the DNA of S*treptococcus pneumoniae* was found with the PCR method in the pleural fluid. Subsequently, the oxygen requirement increased and the patient needed mechanical ventilation. A CT scan was performed and showed a wide area of bilateral lung consolidation and bilateral pleural effusion (Figure 1). 

Five days after the surgical procedure, a new echocardiographic examination demonstrated a large pericardial effusion despite the presence of the drain. A second surgical procedure was performed with a lower J ministernotomy. A pleuropericardial window was created on the right side and the pericardial collection was removed with difficulty because it was dense, friable and partly consolidated. 

**Figure 2:**
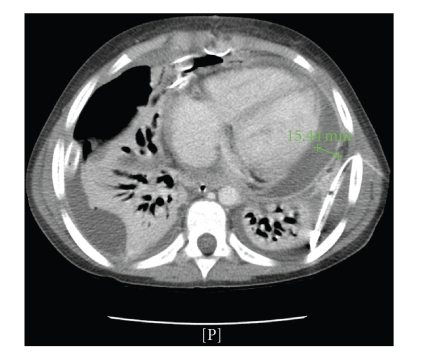
CT scan after creation of a pleuropericardial window on the right side.

One week later a new CT scan showed an increased extent of the lung consolidation, an increased amount of pleural effusion on the right side, and a mild increase in pericardial effusion (Figure 2). 

Urokinase was administered intrapleurally and intrapericardially for three days with significant improvement of chest X-ray. However, in the subsequent days further accumulation of pericardial effusion with clinical signs of tamponade occurred and further surgery was deemed necessary. A full sternotomy was performed and the pericardial sac was removed from phrenic nerve to phrenic nerve. Both pleural spaces were explored and fully drained. Antibiotic treatment was started with fluconazole, linezolid, amikacin, piperacillin, and tazobactam, and 15 gr. of immunoglobuline was administered. 

A progressive improvement of the general conditions lead to extubation within two weeks with a marked reduction of leucocytosis (6,94 × 10^3^/mmc), CRP (0,3 mg/dL), procalcitonine (0,25 ng/mL), and NT-proBNP (471 pg/mL). One month later the patient was discharged. 

At three-month follow-up, there was no evidence of residual or recurrent pleural or pericardial effusion and the patient was back to normal life.

## 2. Discussion

Bacterial pneumonia is a frequent infectious disease in children and it could be presented in association with pleural effusion [1]. The effusion is usually exudative and occurs in up to 60% of patients admitted with pneumonia [2], and it could be the result of a progression of the parenchimal disease [3]. 

The association between pleural effusion and pericardial effusion has been described in 23% of patients with complicated pneumonia [4] and in up to 54% of patients with parapneumonic effusion [3]. A possible explanation is related to a sympathetic pericardial effusion secondary to an adjacent infectious process [3] or is amenable to the involvement of common lymphatic channels in the left hemi-thorax draining the left pleural cavity and the pericardial space. In effect Li [5] described a connection between parietal pleural lymphatics and the pleural spaces via stomas and Riquet et al. [6] demonstrated that drainage of the pericardial lymphatic vessels was mainly directed toward the tracheobronchial nodes and prepericardial nodes. Pericardial effusions are generally of small entity and usually disappear under treatment of the infectious underlying lung disease. In our case there was a bilateral involvement of the pleural spaces and the pericardial effusion was not only causing a cardiac tamponade but was purulent as well. The finding of thickened pericardium and the chemical examination of the fluid drained suggested a purulent pericarditis process, an exceptionally rare condition in children. Even more rare is cardiac tamponade following a pleuropericardial involvement of a lung infection. Al-Sabbagh et al. [7] reported a similar case which resolve with an ultrasound-guided insertion of a subxifoid pericardial catheter. 

## 3. Conclusion

We describe a case of pleuropericardial effusion complicating a bacterial pneumonia. In our case the pericardial fluid density and the recurrence of effusion causing cardiac tamponade required the complete removal of the pericardial sac while the conventional pleuropericardial window and subxifoid approach to pericardial effusion revealed their inefficacy. The more aggressive approach was the only way to gain a stable hemodynamic and to resolve the infectious disease. After total pericardiectomy the effusion disappeared completely and the infectious process progressively disappeared.
